# 

**Published:** 1884-11

**Authors:** 


### The Ninth Meeting of the International Medical Congress.

The next meeting of this body will be held in August, 1887, in Washington City. The place of holding these meetings is not determined by the body itself, but mainly by invitation; any country or locality can extend an invitation, and from those given, the committee decides upon the one offering the most favorable inducements. It was supposed that Berlin would be the next place of meeting, but notwithstanding Professor Virchow pleaded for Berlin, he presented no special invitation, as he and his colleagues were under the impression that the Congress itself decides its place of meeting. On some accounts we think that Berlin would have been a better place, and in one sense was really entitled to the meeting. We have not seen the proceedings of the recent Congress, nor any special synopsis of them, and are not aware that anything was done in dental matters. What shall be done in this direction by the next Congress is undoubtedly in the hands of the profession of this country, and it well be well to give attention to this at an early date, in order that, if a section of dentistry is placed upon the programme, ample time may be afforded for thorough preparation. The committee having in charge the organization, to be held in Washington City, is as follows: Drs. Austen Flint, New York; I. Minus Hayes, Philadelphia; Lewis Sayer, New York; Christopher Johnson, Baltimore; George J. Engleman, St. Louis; J. S. Brown, U. S. Navy; J. S. Billings, U. S. Army. We call attention to this matter early, so that that the profession may have it in mind, and embrace the opportunity to do that which is for the best interests of the profession.

### North Western Dental Association.

A special meeting of this body will be held in the rooms of the Y. M. C. A., at Morehead, Minnesota, on Tuesday and Wednesday, November the 18th and 19th, 1884. It is hoped that the members of the profession in that region will be present at the meeting, and do what they may to promote its interest. Provision has been made for a good meeting; special rates have been secured at the J. Cook and Grand Pacific Hotels. The St. P. & M. M. Railroad will return those present at one-fifth fare. The Fargo Southern R. R. will sell round-trip tickets at one and one-fifth rate. All dentists on that road, intending to be present, can receive their certificate by notifying the Secretary, Dr. S. J. Hill, Fargo, Dakota Territory, at any time before the meeting. Measures will be taken at this meeting with a view to secure a law, this winter, regulating the practice of dentistry in the Territory. This good work should have the cooperation of every dentist of any influence in the Territory, and of every well-wisher of his profession. Let all go prepared to take part in the work of the meeting.

### THE DENTAL REGISTER,

A MONTHLY MAGAZINE DEVOTED TO THE INTERESTS OF THE

### DENTAL PROFESSION.

*Terms Reduced to $2.00 Per Year. Single Copies, 25 Cents.*

**EDITED BY PROF. J. TAFT, D.D.S.**

—AND—

**Published by SPENCER & CROCKER, Ohio Dental and Surgical Depot.**

The volume commences in January and closes in December.

The REGISTER being issued monthly is always fresh, therefore Indispensable to the practitioner who desires to keep posted up with the new inventions and improvements which are daily developed. Neither expense nor effort will be spared to give value and variety to its contents Articles will be illustrated with well executed engravings whenever they will add to the interest or assist to explanation.

Its extensive circulation and frequency of issue make it one of the BEST DENTAL ADVERTISING MEDIUMS in the United States.

All subscriptions must begin with January or July.

Local or special notices, five lines or less, will be inserted for $3 each insertion; over five lines, 50 cts. per line.

All advertising bills payable in advance, or at furthest, after the issue of the first number containing the advertisement. All transient advertisements to be paid for in advance.

☞CONTRIBUTIONS SOLICITED.

Exclusively Editorial Communications should be addressed to the Editor.

The latest number of the REGISTER may be ordered with or without other goods at any time, and all letters should be addressed to

